# Methanol-Promoted Lipid Remodelling during Cooling Sustains Cryopreservation Survival of *Chlamydomonas reinhardtii*

**DOI:** 10.1371/journal.pone.0146255

**Published:** 2016-01-05

**Authors:** Duanpeng Yang, Weiqi Li

**Affiliations:** 1 Key Laboratory for Plant Diversity and Biogeography of East Asia, Kunming Institute of Botany, Chinese Academy of Sciences, Kunming, China; 2 Germplasm Bank of Wild Species, Kunming Institute of Botany, Chinese Academy of Sciences, Kunming, China; 3 University of Chinese Academy of Sciences, Beijing, China; Arizona State University, UNITED STATES

## Abstract

Cryogenic treatments and cryoprotective agents (CPAs) determine the survival rate of organisms that undergo cryopreservation, but their mechanisms of operation have not yet been characterised adequately. In particular, the way in which membrane lipids respond to cryogenic treatments and CPAs is unknown. We developed comparative profiles of the changes in membrane lipids among cryogenic treatments and between the CPAs dimethyl sulfoxide (DMSO) and methanol (MeOH) for the green alga *Chlamydomonas reinhardtii*. We found that freezing in liquid nitrogen led to a dramatic degradation of lipids, and that thawing at warm temperature (35°C) induced lipid remodelling. DMSO did not protect membranes, but MeOH significantly attenuated lipid degradation. The presence of MeOH during cooling (from 25°C to −55°C at a rate of 1°C/min) sustained the lipid composition to the extent that membrane integrity was maintained; this phenomenon accounts for successful cryopreservation. An increase in monogalactosyldiacylglycerol and a decrease in diacylglycerol were the major changes in lipid composition associated with survival rate, but there was no transformation between these lipid classes. Phospholipase D-mediated phosphatidic acid was not involved in freezing-induced lipid metabolism in *C*. *reinhardtii*. Lipid unsaturation changed, and the patterns of change depended on the cryogenic treatment. Our results provide new insights into the cryopreservation of, and the lipid metabolism in, algae.

## Introduction

Cryopreservation is commonly used for storing viable cells, tissues, organs or organisms at ultralow temperatures, usually involving immersion in liquid nitrogen at −196°C. Using this procedure, organisms can be preserved with their morphological, physiological, biochemical and genetic properties unchanged [1]. As a consequence of the development of cryopreservation, cryobanking was established as a means of protecting biodiversity, important (valuable or endangered) organisms and genetic resources [2]. The methods of cryopreservation for plants include two-step cooling: vitrification and encapsulation-dehydration [3]. Herein, we focus on two-step cooling. When using this method, materials are first cooled with a cryoprotective agent (CPA) at a slow and constant rate (0.2–1°C/min) to between −35°C and −75°C; then they are dipped into liquid nitrogen for storage [4]. Two-step cooling is an effective method for cryopreserving *Chlamydomonas reinhardtii*, which is an important model organism; the viability on revival is usually >40% [4]. The use of a CPA is essential for successful cryopreservation. Such agents include methanol (MeOH) and dimethyl sulfoxide (DMSO). MeOH is usually used when cryopreserving freshwater and terrestrial algal strains, whereas DMSO is more effective than MeOH for cryopreserving marine algae [5]. DMSO is a good CPA for higher plants, but CPA that contains 2–10% MeOH works well for algae. Cryopreservation of *C*. *reinhardtii* is also important for industrial applications [6]. The cryopreservation of this organism has been documented intensively, in terms of cryoprocedure and the choice of CPA [2]. However, despite the importance of cryopreservation and the attention that has been paid to which methods are effective, little is known about the cellular changes that cryopreserved organisms undergo and how these changes sustain survival.

Cryopreservation is a complicated process, in which ice nucleation and glass transition are two determinative events for the survival of cryopreserved organisms. In most biological systems, the temperature of homogeneous ice nucleation is at, or around, −40°C [7]. At this temperature, water molecules form an “ice embryo” of a critical size that then grows into a crystal. The glass transition temperature is around −130°C to −137°C. At this temperature the “glass state” is formed, called vitrification, in which the crystals solidify together [8]. The ice nucleation and glass transition can be modulated by cryoprotective strategies, mainly by changing the CPA and cooling rate. In general, an effective CPA should penetrate the cell and be nontoxic at its working concentration [2]. Glycerol, DMSO, low molecular weight sugars and MeOH are common cell-penetrating cryoprotectants used for higher plants, algae and cyanobacteria [2]. However, different CPAs have different capacity to permeate cellular membranes and different organisms differ in the permeability of their membranes. For example, MeOH can permeate the *Chlorococcum texanum* membrane faster than water and DMSO slower than water [9]. The differences in CPA permeation and membrane permeability result in different survival rates of cells of different organisms. For example, treating *C*. *reinhardtii* with DMSO overnight then freezing in liquid nitrogen yields a low survival rate of cells [10]. It will be evident from the foregoing that knowing the responses of membranes to different CPAs is important for successful cryopreservation.

In addition to the issues raised for survival by permeability, the fluidity and integrity of membranes play important roles in cells' resistance to low temperature [11, 12]. Cells change their lipid compositions to maintain their membrane fluidity and integrity. To adapt to above-zero low temperature, *C*. *reinhardtii* reduces its total lipid content but increases the lipid desaturation [13]. At subzero low temperatures, lesions form in membranes and damage their integrity [14, 15]. Different types of lesion form in different temperature ranges. At −2°C to −4°C, an expansion-induced lysis occurs. At temperatures between −4°C and −10°C, a hexagonal II phase forms. At temperatures below −10°C, other types of membrane lesions form as the consequence of lowered water potential and severe dehydration. The suppression of phosphatidic acid (PA), which is a lipid component that favours the formation of a hexagonal II phase, improves freezing-tolerance in *Arabidopsis thaliana* [16]. During cryopreservation, cells experience chilling (10°C to 0°C), freezing (from 0°C to −20°C), deep freezing (below −20°C) and thawing (from −196°C to 35°C in minutes). However, the way in which membrane composition changes during these processes, and how these changes contribute to cryopreservation, remains unclear.

*C*. *reinhardtii* membranes consist mainly of glycerolipids. These include (a) seven classes of phospholipids: diacylglycerol (DAG), diacylglyceryl-N,N,N-trimethylhomoserine (DGTS), phosphatidylethanolamine (PE), phosphatidylinositol (PI), phosphatidylserine (PS), phosphatidic acid (PA), and phosphatidylglycerol (PG); and (b) two classes of plastidic galactolipids: monogalactosyldiacylglycerol (MGDG) and digalactosyldiacylglycerol (DGDG) [17–19]. The lipid components of *C*. *reinhardtii* are the same as those in higher plants, with the exception of DGTS substituting for phosphatidylcholine (PC). In higher plants, PA and DAG are intermediates in lipid metabolism [20]. Abiotic stresses such as cold and dehydration, and senescence-induced degradation of membrane lipids, cause lipids to breakdown. The components go into the PA and DAG pools. As a result, there is a temporary rise in PA and DAG levels [21–24]. The increases in PA are mainly from phospholipase D (PLD)-mediated hydrolysis of phospholipids [25]. In *C*. *reinhardtii*, DAG is a central intermediate in lipid synthesis as it is stored as lipid triacylglycerol (TAG) [26]. An absence of nitrogen from the culture medium leads to the transformation of MGDG into TAG via DAG [6]. A decrease in MGDG and an increase in saturated and monounsaturated fatty acids occur in response to iron starvation [27]. However, the mechanism by which *C*. *reinhardtii* metabolises lipids during cryopreservation is not known.

Most cultivable cyanobacteria, soil microalgae and marine diatoms can be cryopreserved with fairly high viability. Many freshwater and marine eukaryotic algae can also be cryopreserved, but with lower viability [5]. Dinoflagellates, cryptophytes, synurophytes and raphidophytes cannot be cryopreserved [5]. *C*. *reinhardtii*, a unicellular green alga, is called “green yeast” because it is easy to culture and is used extensively in many aspects of cell biology. The genome sequence of *C*. *reinhardtii* has been completed, its genetic map is well-established, and the organism is susceptible to three genetic transformations (nuclear, mitochondrial and chloroplast). These characteristics have made *C*. *reinhardtii* a primary model system [28]. The procedure by which *C*. *reinhardtii* is cryopreserved is well-established [4] and is used in collections worldwide for many different strains [28]. In this study, we used *C*. *reinhardtii* as the test organism to explore molecular changes in membrane lipids during two-step cooling cryopreservation in different CPAs. We found that remarkable lipid changes occurred after cryopreservation, but that each step of cryopreservation had its own effect on lipid composition. DMSO and MeOH have totally different effects on lipid composition. Only combining the effects of cooling and MeOH could sustain survival through cryopreservation. The roles of the lipid classes MGDG, DAG and PA were characterised and the stoichiometric turnover of lipid molecular species was examined.

## Results

### Experimental Design and the Effects of CPAs on the Survival of *C*. *reinhardtii*

**Fig 1:**
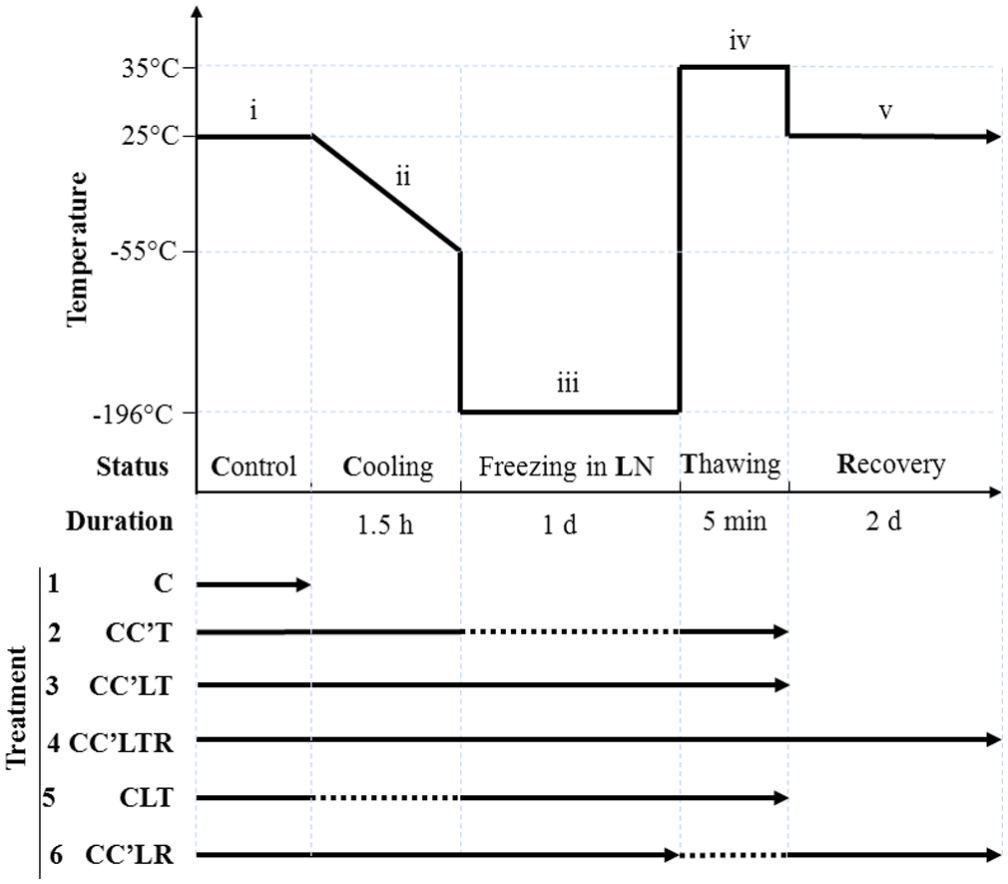
Cryogenic treatments and cryopreservation processes of *C*. *reinhardtii*. The process was divided into five stages: (i) control (normal growth) (C); (ii) cooling from 25°C to −55°C at a rate of 1°C/min (C'); (iii) freezing in liquid nitrogen (L); (iv) thawing at 35°C for 5 min (T); and (v) recovery growth for 2 days in normal growth conditions (R). The processes that the organism underwent in each treatment are indicated with a solid line; for an omitted step, a dotted line is used. There were six different treatment combinations: (1) C: Control (normal growth) only; (2) CC’T: Control, Cooling and Thawing; (3) CC’LT: Control, Cooling, Freezing in liquid nitrogen (LN) and Thawing; (4) CC’LTR: Control, Cooling, Freezing in LN, Thawing and Recovery growth; (5) CLT: Control, Freezing in LN and Thawing; (6) CC’LR: Control, Cooling, Freezing in LN, Incubation at room temperature for 30 min and Recovery growth.

Two-step cooling [4] was performed to cryopreserve *C*. *reinhardtii* in either culture medium, or in culture medium supplemented with either DMSO or MeOH. The processes of cryopreservation and the corresponding survival rates of *C*. *reinhardtii* are illustrated in Fig 1 and Table 1, respectively. During the experimental process, *C*. *reinhardtii* was placed in up to five conditions in the following order (Fig 1): (i) control (normal growth) (C); (ii) cooling from 25°C to −55°C at a rate of 1°C/min (C'); (iii) rapid freezing in liquid nitrogen (L); (iv) thawing at 35°C for 5 min (T); and (v) recovery growth for 2 days in normal growth conditions (R). It is not possible to isolate *C*. *reinhardtii* from its culture medium unless the medium is liquefied, and cells must be treated immediately with hot (75°C) isopropanol to stop any lipid metabolism [16]; hence, conditions i, iv and v were suitable for taking samples.

**Table 1:** Survival rate of *C*. *reinhardtii* in culture medium, culture medium plus 5% DMSO, and culture medium plus 5% MeOH after cryogenic treatments.

Treatment No.	Cryogenic Treatment	Survival rate (%)		
		CM	DMSO	MeOH
1	C	> 90	> 90	> 90
2	CC’T	0	0	75.9 ± 2.5
3	CC’LT	0	0	41.3 ± 2.7
4	CC’LTR	0	0	> 41.3 ± 2.7
5	CLT	0	0	0
6	CC’LR	0	0	> 24.2 ± 3.0

CM, culture medium; DMSO, culture medium plus 5% DMSO; MeOH, culture medium plus 5% MeOH. Values are means ± standard deviation (*n* = 5).

Six treatments, involving different combinations of the conditions (i)—(v) (Fig 1), were used to compare the effects of individual processes on the overall cryopreservation: (1) Control (normal growth) only (C); (2) Control, Cooling and Thawing (CC'T); (3) Control, Cooling, Freezing in liquid nitrogen (LN) and Thawing (CC'LT); (4) Control, Cooling, Freezing in LN, Thawing and Recovery (CC'LTR); (5) Control, Freezing in LN and Thawing (CLT); (6) Control, Cooling, Freezing in LN, Incubation at room temperature for 30 min and Recovery (CC’LR). The corresponding survival rates of *C*. *reinhardtii* in each treatment were investigated in three CPAs: culture medium, culture medium plus 5% DMSO, and culture medium plus 5% MeOH (Table 1). For example, comparison between treatments 2 and 3 showed the effects of liquid nitrogen freezing, while comparison between treatments 3 and 5 showed the effects of cooling on cryopreservation.

We found that no *C*. *reinhardtii* survived after cryogenic treatments in either culture medium or culture medium plus 5% DMSO (Table 1), which means that DMSO had no cryoprotective effects. This finding is consistent with those reported previously [4]. In contrast, the *C*. *reinhardtii* survival rate was significant in culture medium plus 5% MeOH. The survival rates in culture medium plus 5% MeOH varied between different treatments. For example, comparing treatment CC’T with the control, cooling resulted in a fall in survival rate of more than 14% (from >90.0% to 75.9% ± 2.5%) (Table 1). Comparing treatment CC’LT with treatment CC’T, freezing in liquid nitrogen resulted in a fall in survival rate of 34% (from 75.9% ± 2.5% to 41.3% ± 2.7%). Most importantly, without the cooling step, i.e. cooling from 25°C to −55°C at a rate of 1°C/min, no *C*. *reinhardtii* survived. These results indicate that both MeOH and cooling are necessary for successful cryopreservation of *C*. *reinhardtii* and that when the culture medium contains 5% MeOH, individual parts of the cryopreservation process make their own contributions to the survival level of the alga.

### Lipid Profiling and Overall Changes in Lipid Composition during Cryogenic Processes

**Fig 2:**
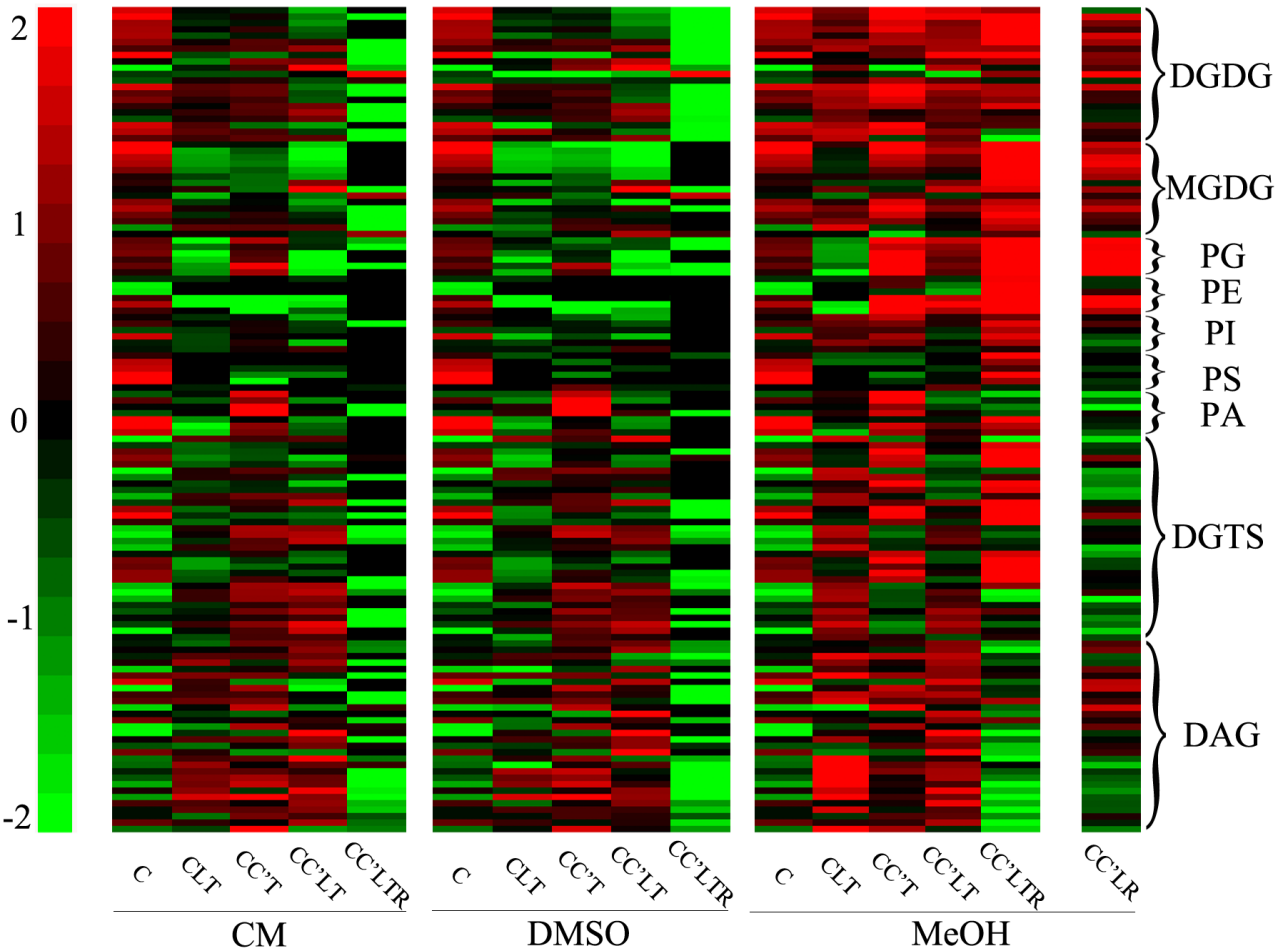
Heatmap of lipid classes detected following cryogenic treatments. The labels below the images show the cryogenic treatments used and the CPA added: CM, culture medium; DMSO, culture medium plus 5% DMSO; MeOH, culture medium plus 5% MeOH. The right-hand labels show lipid classes: DGDG, digalactosyldiacylglycerol; MGDG, monogalactosyldiacylglycerol; PG, phosphatidylglycerol; PE, phosphatidylethanolamine; PI, phosphatidylinositol; PS, phosphatidylserine; PA, phosphatidic acid; DGTS, diacylglyceryl-N,N,N-trimethylhomoserine; DAG, diacylglycerol. The colour of each bar indicates the relative abundance of each lipid molecule, shown as relative variation compared with the mean centre of each lipid species in all treatments. Values are means (*n* = 4 or 5).

**Fig 3:**
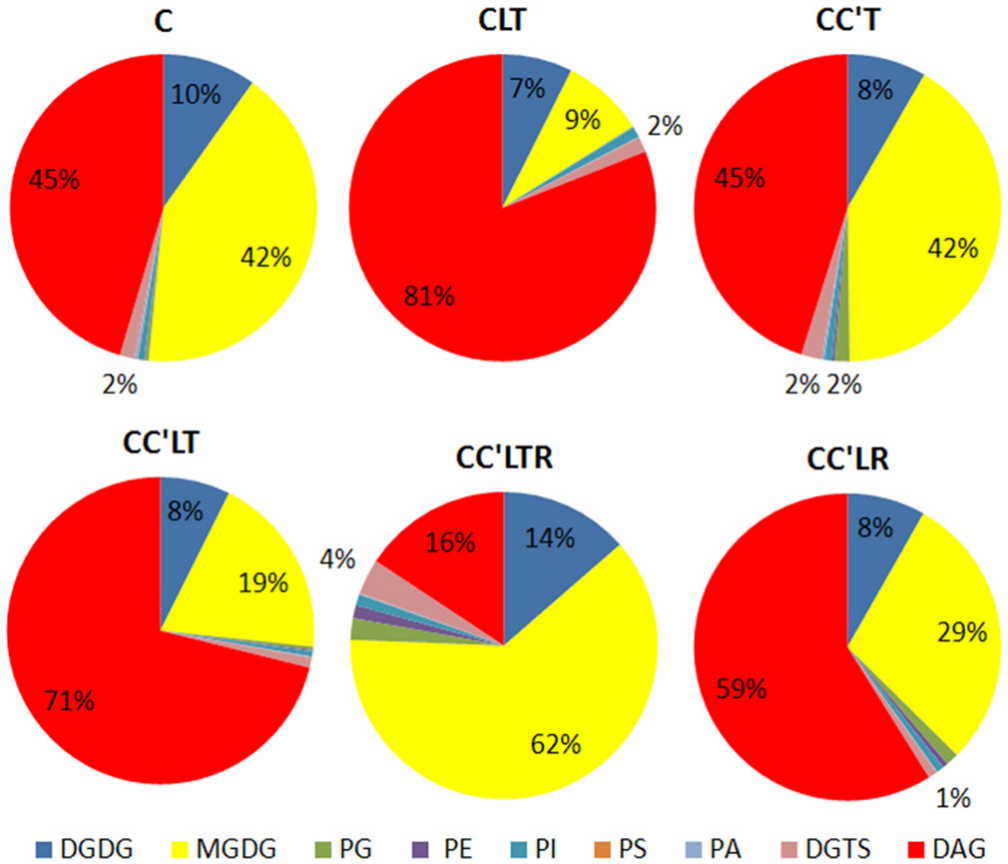
Molar percentages of lipid classes in *C*. *reinhardtii* after cryogenic treatments. Values are means (*n* = 4 or 5).

**Fig 4:**
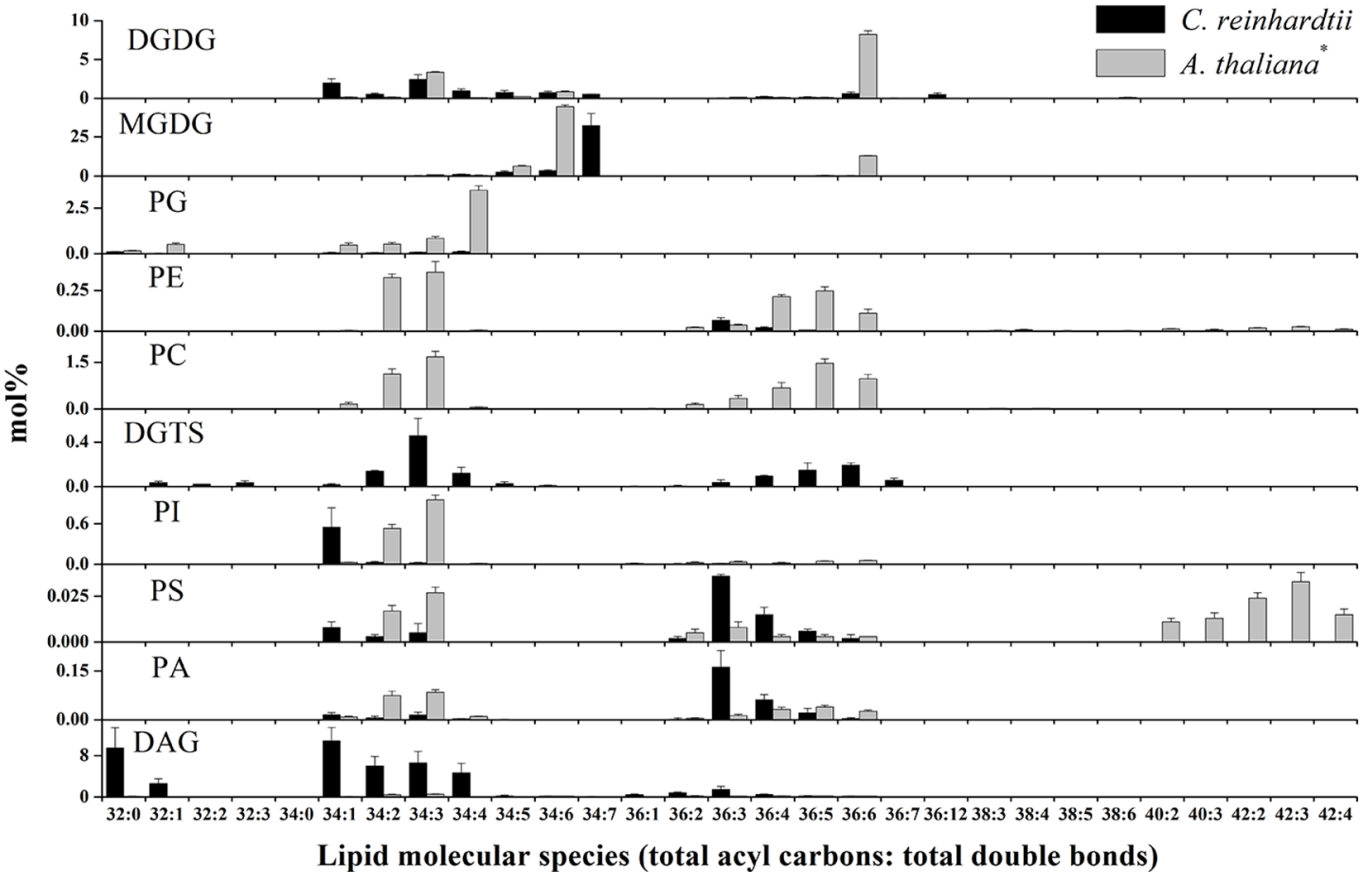
Composition of lipid classes in *C*. *reinhardtii* and *A*. *thaliana* in normal growth conditions. Lipid data for *A*. *thaliana* are from Li et al. (2014). Values are means ± standard deviation (*n* = 4 or 5).

To explore the changes in membrane lipids and how they affect cryopreservation, we harvested *C*. *reinhardtii* that were subjected to various cryogenic processes (Fig 1) in culture medium only, culture medium plus 5% DSMO, or culture medium plus 5% MeOH (Table 1), then we isolated and analysed their membrane lipids. We identified 210 molecular species belonging to nine lipid head-group classes (MGDG, DGDG, PG, PE, PI, PS, PA, DAG and DGTS) and quantified their levels (nmol per 10 million cells) (similarly hereafter) (Fig 2). The total levels and percentage composition (mol%) of each class are shown in Table 2 and Fig 3. We found that the composition of membrane lipids in *C*. *reinhardtii* differs from that in higher plants, for example *A*. *thaliana* (Fig 4). The differences pertained not only to the substitution of DGTS for PC, as expected [17], but also to the molecular constituents of DAG and MGDG. In normal growth conditions, *C*. *reinhardtii* contained 32:0 (total acyl carbons:total double bonds), 32:1, 34:1, 34:2, 34:3, 34:4, 36:1, 36:2, 36:3, 36:4 DAG, and 34:7 MGDG, of which *A*. *thaliana* had either none or very little. The most abundant membrane lipids in *C*. *reinhardtii* were MGDG, DAG, and DGDG, but in *A*. *thaliana* they were MGDG, DGDG, PC and PG. These results suggest that DAG has important functions in determining the properties of the membrane and in lipid metabolism in *C*. *reinhardtii*. Given that lipids are fundamental contributors to membrane function and that DAG and MGDG are distributed mainly on extraplastidic and plastidic membranes, respectively, *C*. *reinhardtii* may have distinct features from *A*. *thaliana* in both membrane composition and lipid metabolism.

**Table 2:** Levels of lipid classes in *C*. *reinhardtii* after cryogenic treatments in culture medium, culture medium plus 5% DMSO, and culture medium plus 5% MeOH.

Lipid class	CPA type	Lipid (nmol per 10 million cells)					
		C	CLT	CC’T	CC’LT	CC’LTR	CC’LR
DGDG	CM	6.20 ± 0.49^a^	2.90 ± 0.24^c^	3.50 ± 0.25^b^	3.35 ± 0.16^b^	0.24 ± 0.04^d^	-
DGDG	DMSO	6.20 ± 0.49^a^	2.72 ± 0.43^c^	3.12 ± 0.41^bc^	3.41 ± 0.3^b^	0.26 ± 0.02^d^	-
DGDG	MeOH	6.20 ± 0.49^bc^	5.46 ± 0.66^c^	7.93 ± 1.52^b^	6.52 ± 0.86^bc^	16.65 ± 4.12^a^	5.66 ± 1.75^c^
MGDG	CM	26.68 ± 1.96^a^	3.17 ± 0.13^c^	4.66 ± 0.65^b^	1.93 ± 0.35^d^	0.03 ± 0.01^e^	-
MGDG	DMSO	26.68 ± 1.96^a^	2.61 ± 0.12^bc^	3.09 ± 0.66^b^	1.77 ± 0.28^c^	0.04 ± 0.01^d^	-
MGDG	MeOH	26.68 ± 1.96^c^	6.88 ± 0.45^e^	39.34 ± 3.26^b^	16.97 ± 2.92^d^	79.78 ± 5.74^a^	18.98 ± 3.35^d^
PG	CM	0.30 ± 0.02^a^	0.07 ± 0.03^b^	0.37 ± 0.23^a^	0.06 ± 0.03^b^	0.01 ± 0.01^c^	-
PG	DMSO	0.30 ± 0.02^a^	0.09 ± 0.04^c^	0.20 ± 0.06^b^	0.06 ± 0.02^c^	0.02 ± 0.01^d^	-
PG	MeOH	0.30 ± 0.02^c^	0.07 ± 0.03^d^	1.48 ± 1.01^b^	0.33 ± 0.09^c^	2.89 ± 1.58^a^	0.91 ± 0.53^bc^
PE	CM	0.08 ± 0.01^a^	0.01 ± 0^b^	0.01 ± 0^b^	0.01 ± 0^b^	0^c^	-
PE	DMSO	0.08 ± 0.01^a^	0.01 ± 0^b^	0^b^	0.01 ± 0^b^	0^b^	-
PE	MeOH	0.08 ± 0.01^c^	0.01 ± 0^d^	0.28 ± 0.24^bc^	0.13 ± 0.03^c^	1.67 ± 0.54^a^	0.32 ± 0.14^b^
PI	CM	0.41 ± 0.15^b^	0.42 ± 0.16^b^	0.63 ± 0.15^a^	0.42 ± 0.08^b^	0^c^	-
PI	DMSO	0.41 ± 0.15^a^	0.38 ± 0.16^a^	0.40 ± 0.27^a^	0.48 ± 0.08^a^	0^b^	-
PI	MeOH	0.41 ± 0.15^c^	0.81 ± 0.09^b^	0.83 ± 0.21^b^	0.47 ± 0.19^c^	1.40 ± 0.39^a^	0.57 ± 0.32^bc^
PS	CM	0.05 ± 0.01^a^	0^b^	0^b^	0.01 ± 0^b^	0^b^	-
PS	DMSO	0.05 ± 0.01^a^	0.01 ± 0.01^b^	0.01 ± 0^b^	0.01 ± 0^b^	0^b^	-
PS	MeOH	0.05 ± 0.01^a^	0.01 ± 0^c^	0.01 ± 0^c^	0.02 ± 0.01^c^	0.04 ± 0.02^b^	0.01 ± 0^c^
PA	CM	0.19 ± 0.05^a^	0.04 ± 0.02^b^	0.20 ± 0.03^a^	0.05 ± 0.01^b^	0^c^	-
PA	DMSO	0.19 ± 0.05^a^	0.06 ± 0.03^b^	0.21 ± 0.02^a^	0.06 ± 0.01^b^	0^c^	-
PA	MeOH	0.19 ± 0.05^ab^	0.05 ± 0.03^d^	0.23 ± 0.12^a^	0.09 ± 0.01^cd^	0.13 ± 0.05^bc^	0.05 ± 0.02^d^
DAG	CM	24.39 ± 5.15^c^	37.28 ± 4.47^b^	55.82 ± 3.07^a^	56.73 ± 6.71^a^	14.67 ± 1.79^d^	-
DAG	DMSO	24.39 ± 5.15^d^	35.65 ± 4.25^c^	51.99 ± 10.63^b^	62.37 ± 9.34^a^	12.65 ± 7.83^e^	-
DAG	MeOH	24.39 ± 5.15^c^	65.42 ± 20.05^a^	43.59 ± 3.83^b^	63.09 ± 8.03^a^	19.04 ± 1.85^c^	40.12 ± 6.22^b^
DGTS*	CM	0.98 ± 0.27^a^	0.65 ± 0.11^b^	1.00 ± 0.21^a^	1.06 ± 0.21^a^	0.04 ± 0.01^c^	-
DGTS*	DMSO	0.98 ± 0.27^a^	0.58 ± 0.05^b^	1.11 ± 0.43^a^	1.04 ± 0.20^a^	0.05 ± 0.01^c^	-
DGTS*	MeOH	0.98 ± 0.27^c^	1.27 ± 0.12^bc^	2.10 ± 0.87^b^	0.92 ± 0.22^c^	4.59 ± 2.00^a^	0.65 ± 0.18^c^
Total	CM	58.44 ± 4.75^b^	43.44 ± 4.44^c^	65.30 ± 2.28^a^	62.06 ± 7.79^ab^	15.00 ± 1.81^d^	
Total	DMSO	58.44 ± 4.75^b^	41.67 ± 4.07^c^	59.09±11.72^ab^	68.36 ± 9.31^a^	12.93 ± 7.88^d^	
Total	MeOH	58.44 ± 4.75^e^	77.64±17.34^cd^	93.74 ± 5.22^b^	87.85 ± 9.77^bc^	124.95 ± 7.54^a^	66.76±10.63^de^

CM, culture medium; DMSO, culture medium plus 5% DMSO; MeOH, culture medium plus 5% MeOH. Values are means ± standard deviation (*n* = 4 or 5). Different superscript letters indicate that values in a row are significantly different (*P* < 0.05).

* DGTS was quantified by PC standard.

Figs 2 and 3 show that the levels and compositions of lipids in *C*. *reinhardtii* differed remarkably depending on the cryogenic processes used. For example, the levels of most lipids decreased in both culture medium and culture medium with 5% DMSO as the CPA; in contrast, the levels of most lipids increased in culture medium with 5% MeOH after cryogenic treatments. These observations indicate that lipids change during cryogenic processes and that different lipids have distinct patterns of change. These findings suggest that individual lipids have their own roles to play in cryopreservation and that cryoprotectants have specific effects on membrane integrity during cryopreservation. A detailed analysis is presented below.

### The Degradation of Membrane Lipids during Cryogenic Processes in Culture Medium Only and Culture Medium plus 5% DMSO

In culture medium only, the levels of all membrane lipids of *C*. *reinhardtii* (except for DAG) fell significantly upon either cooling to −55°C or freezing in liquid nitrogen. For example, the level of DGDG decreased from 6.2 (C) to 2.9 after freezing in liquid nitrogen and thawing (CLT; Table 2); to 3.5 after cooling and thawing (CC’T); and to 0.24 after recovery growth for 2 days after cooling, freezing in liquid nitrogen, and thawing (CC’LTR; Table 2). Levels of MGDG, PG, PI, PS, PA, and DGTS fell to almost zero after CC’LTR. These results indicate that massive lipid degradation took place and that membranes deteriorated in *C*. *reinhardtii* on cryogenic treatments. These phenomena explain the zero survival rate on cryopreservation in culture medium alone (Table 1). The levels of lipid decrease, the manner in which they decreased, and the changes in lipid composition, were the same in culture medium plus 5% DMSO as in culture medium alone (Table 2; Fig 3). These results show that DMSO plays no role in the protection of *C*. *reinhardtii* membranes, which may be the reason that DMSO has no cryoprotective effect (Table 1).

### Changes of DAG and PA during Cryogenic Processes in Culture Medium Alone and Culture Medium plus 5% DMSO

The increases in DAG corresponded to decreases in other lipid classes in all cryogenic processes (Table 2). For example, in culture medium alone, DAG increased from 24.39 in the control to 37.28 under CLT, to 55.82 under CC’T, and to 56.73 under CC’TL. Other lipids showed a general trend of decreasing levels. DAG decreased from 24.9 to 14.67 on recovery growth for 2 days after thawing (i.e. CC'LTR). Given that DAG is an intermediate in lipid metabolism [29] and the levels of the DAG pool are determined by the amounts of DAG both produced and consumed, these results suggest that the freezing-induced degradation of membrane lipids is likely to occur through a DAG intermediate in *C*. *reinhardtii*. To determine the role that DAG plays as an intermediate, we examined the turnover of molecular species between DAG and other lipids by comparing treatment C with CC'LT (Table 3). Surprisingly, the increases in DAG did not correspond stoichiometrically to the decreases in other lipids. For example, the 32:0 DAG level increased to 5.393 but other 32:0 lipids hardly changed at all. These results indicate that the degradation of lipids did not, at least directly, occur via a DAG intermediate during cryogen-induced membrane deterioration.

**Table 3:** Stoichiometric comparison of molecular species of lipids between C and CC’LT treatments with culture medium only, culture medium plus 5% DMSO, or culture medium plus 5% MeOH as the CPA.

Molecular species	Change between C and CC’LT (nmol per 10 million cells)					
	CM		DMSO		MeOH	
	DAG	Others	DAG	Others	DAG	MGDG
32:0	5.393	-0.038	6.506	-0.043	5.304	-
32:1	1.531	-0.031	1.459	-0.039	1.873	-
34:1	6.047	0.801	8.839	0.844	9.526	0.077
34:2	16.196	-0.148	16.862	-0.199	13.578	0.073
34:3	-0.540	-1.587	-0.216	-1.732	2.238	-0.049
34:4	-1.030	-0.897	-0.826	-1.250	1.147	-0.283
34:5	0.223	-1.869	0.128	-2.003	0.232	-0.583
34:6	0.127	-2.433	-0.061	-2.492	0.264	-0.853
34:7	0.080	-20.382	0.108	-20.691	0.081	-7.963
34:8	0.009	-0.090	0.008	-0.095	0.014	-0.047
34:9	-	0.001	-	0.000	-	-
36:1	0.141	0.080	-0.093	0.063	0.159	0.005
36:2	0.472	0.077	0.136	0.058	0.321	0.005
36:3	2.784	-0.124	2.926	-0.130	2.819	0.011
36:4	1.439	-0.208	0.850	-0.235	1.198	-0.015
36:5	0.277	-0.158	0.094	-0.232	0.462	-0.021
36:6	0.057	-0.587	0.007	-0.546	0.226	-0.048
36:7	-0.004	-0.090	0.014	-0.092	0.014	-0.031
36:8	-	-0.012	-	-0.006	-	-0.001
36:9	-	0.110	-	0.114	-	-
36:10	-	0.007	-	0.015	-	-
36:11	-	0.009	-	0.005	-	-
36:12	-	-0.307	-	-0.307	-	-

CM, culture medium; DMSO, culture medium plus 5% DMSO; MeOH, culture medium plus 5% MeOH. Values are means (*n* = 4 or 5). “-” indicates that the molecular species was below the limit of detection.

PA forms by PLD-catalysed hydrolysis during freezing and thawing and its molar increases correspond to the molar decrease in PC and MGDG in higher plants [21]. However, during cryogenic processes in *C*. *reinhardtii*, PA decreased or did not change (Table 2). Further *in vitro* analysis showed that *C*. *reinhardtii* had no PLD activity (S1 Fig). This evidence suggests that PA is not an intermediate in lipid metabolism and that PLD does not function in lipid metabolism during cryogenic processes in *C*. *reinhardtii*.

### The Effects of MeOH and Cooling on Membrane Lipids

As indicated above, successful cryopreservation of *C*. *reinhardtii* required the combination of MeOH and cooling (i.e., processing from 25°C to −55°C at a rate of 1°C/min). We first analysed the effects of 5% MeOH in culture medium on membrane lipids on CLT treatment because CLT did not involve the cooling step (Fig 1; Table 2). On CLT treatment, the trends in lipid composition were of degradation in the same manner as that which occurred in culture medium plus 5% DMSO. However, the levels of major lipids in culture medium plus 5% MeOH were significantly higher, and other lipids were at least not lower, than those in culture medium or culture medium plus 5% DMSO. For example, DGDG was present at 5.64 in medium plus 5% MeOH, significantly higher than the values 2.72 and 2.90 that were found in culture medium and culture medium plus 5% DMSO, respectively; MGDG was present at 6.88 in culture medium plus 5% MeOH, significantly higher than the values 2.61 and 3.17 that were found in culture medium and culture medium plus 5% DMSO; the total lipid content was 74.64 in culture medium plus 5% MeOH, significantly higher than the values 43.44 and 41.67 that were found in culture medium and culture medium plus 5% DMSO. These data demonstrate that MeOH is able to attenuate lipid degradation.

We then analysed the effects of cooling on membrane lipids by comparing lipids between CLT and CC’LT, because cooling was the only difference between these two treatments. The levels of MGDG, PG, and PE after CC’LT were significantly higher than those after CLT. In particular, MGDG increased by 2.5-fold, from 6.88 to 16.97; the total lipid content increased slightly from 77.64 to 87.85. The levels of DGDG, DAG and DGTS were roughly equal between the two treatments. In terms of the lipid composition, there was a difference of 10% in the MGDG and DAG levels between CLT and CC’LT (Fig 3): with cooling, the amount of MGDG increased from 9% to 19% of the total lipid and that of DAG decreased from 81% to 71%. The evidence indicates that cooling is able to change the lipid composition and favour membrane integrity. All these results show that both MeOH and cooling sustain lipid composition to maintain membrane integrity, and that MGDG and DAG are the lipids most susceptible to MeOH and cooling. *C*. *reinhardtii* cannot survive cryopreservation without both MeOH and cooling; our results also suggest that membranes cannot be maintained unless MeOH and cooling are combined in the cryogenic process.

### The Effects of Freezing in Liquid Nitrogen on Membrane Lipids

We compared the lipids between the treatments CC’T and CC’LT in culture medium plus 5% MeOH to determine the effects on the membranes of freezing in liquid nitrogen (Fig 1). There was no difference in levels of DGDG and PS (Table 2). Levels of MGDG, PG, PE, PI, PA, and DGTS were significantly lower after CC'LT than after CC’T. Levels of DAG after CC’LT were markedly higher than after CC’T. The percentages of MGDG and DAG among the total lipids were 71% and 19% after CC’LT, respectively, whereas they were 40% and 45% after CLT (Fig 3). These decreases in MGDG, PG, PE, PI, PA, and DGTS lipids and the increase in DAG shows that lipid degradation occurred. Thus, freezing in liquid nitrogen causes membrane lipid degradation. Overall, we may conclude that cooling attenuates lipid degradation, whereas freezing in liquid nitrogen causes it.

### The Effects of Thawing on Membrane Lipids and the Membrane Lipids after Recovery Growth

The differences in lipids between the CC’LR and CC’LTR treatments showed the effects of a 35°C thawing step on membranes (Fig 1). The levels of DGDG, MGDG, PG, PI, PE, PS, PA, and DGTS after CC’LR were dramatically lower than those after CC’LTR, and the level of DAG after CC’LR was twice that after CC’LTR in culture medium plus 5% MeOH (Table 2). The total amount of lipids after CC’LR was much lower than after CC’LTR. The percentages of MGDG and DAG among the total lipids were 59% and 29% respectively after CC’LR, whereas they were 16% and 62% after CC’LTR (Fig 3). These data show that *C*. *reinhardtii* experienced significant lipid synthesis in the recovery phase of CC’LTR treatment and that lipids remained degraded on CC’LR treatment, suggesting that rapid thawing at a warm temperature (in this case 35°C) benefits membrane recovery.

It was expected that after having experienced recovery growth for 2 days, cryopreserved *C*. *reinhardtii* would have returned to its normal state (i.e. as seen during the control growth phase). However, its membrane lipids differed significantly from those in the control. *C*. *reinhardtii* after CC’LTR showed higher levels of DGDG, MGDG, PG, PE, PI and DGTS, equal levels of PS and PA, and lower levels of DAG, compared to the control (Table 4). The molar percentages of MGDG and DAG in CC'LTR were 62% and 16%, respectively, as against 42% and 46%, respectively, in the control. These results suggest that *C*. *reinhardtii* undergoes significant lipid synthesis after being revived from cryopreservation.

**Table 4:** Stoichiometric comparison of molecular species of lipids between C and CC’LTR treatments with culture medium plus 5% MeOH as the CPA.

Molecular species	Change between C and CC’LTR in MeOH (nmol per 10 million cells)			
	DAG	DGTS	DAG	MGDG
32:0	-3.692	-	-3.692	-
32:1	-0.745	0.084	-0.745	-
32:2	-	0.044	-	-
32:3	-	0.127	-	-
34:1	-3.534	0.148	-3.534	0.096
34:2	1.958	0.158	1.958	0.294
34:3	1.486	1.246	1.486	1.259
34:4	-0.497	0.362	-0.497	5.982
34:5	0.021	0.050	0.021	10.131
34:6	-0.012	0.033	-0.012	12.851
34:7	-0.056	-	-0.056	21.407
34:8	-0.005	-	-0.005	-
36:1	-0.281	-0.001	-0.281	0.002
36:2	-0.354	0.025	-0.354	0.009
36:3	-0.596	0.065	-0.596	0.038
36:4	-0.254	0.387	-0.254	0.056
36:5	-0.036	0.420	-0.036	0.152
36:6	-0.032	0.369	-0.032	0.071
36:7	-0.007	-	-0.007	0.040

MeOH, culture medium plus 5% MeOH. Values are means (*n* = 4 or 5). “-” indicates that the molecular species was below the limit of detection.

### Changes in Lipid Unsaturation during Cryopreservation and in Culture Medium plus 5% MeOH

**Fig 5:**
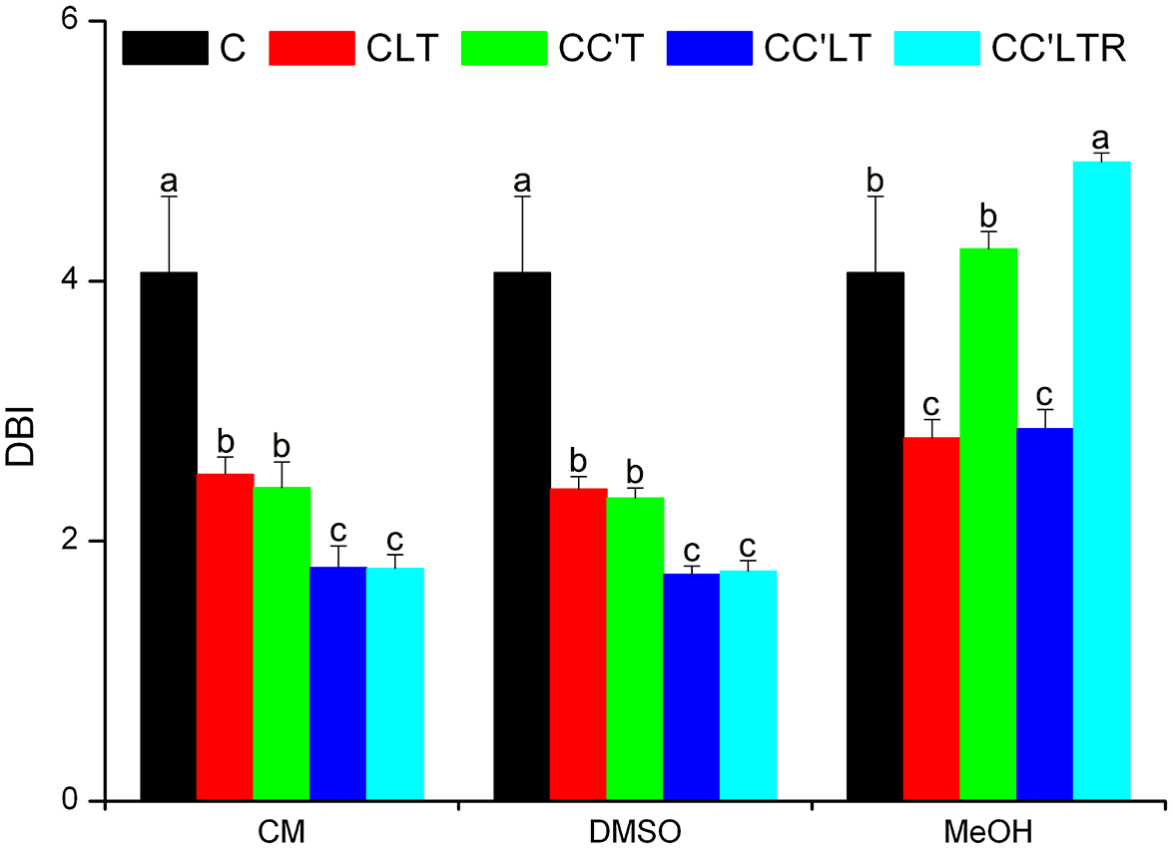
Membrane lipid DBI following cryogenic treatments using culture medium, culture medium plus 5% DMSO, or culture medium plus 5% MeOH as the CPA. CM, culture medium; DMSO, culture medium plus 5% DMSO; MeOH, culture medium plus 5% methanol. Values are means ± standard deviation (*n* = 4 or 5). Values for the same treatment marked with different lowercase letters indicate that the values are significantly different (*P* < 0.05).

We used the double-bond index (DBI) to assess the levels of lipid unsaturation (Fig 5). Cryogenic treatment in culture medium or culture medium plus 5% DMSO resulted in dramatic reductions in the DBI in *C*. *reinhardtii*. The decreases occurred as a result of the massive degradation of lipids. In culture medium plus 5% MeOH, on CLT and CC’LT treatments, lipids had a significantly lower DBI than that in control-treated cells (C). However, the DBI on CC’T and CC’LTR treatments was the same as and higher than that in the control (C), respectively. This means that the lipid unsaturation of *C*. *reinhardtii* depended on the cryogenic treatment. Considering that 1) MGDGs were low after both CLT and CC’LT and high after both CC’T and CC’LTR, and 2) MGDG in *C*. *reinhardtii* contains more double bonds (34:7 MGDG was the highest lipid component (Fig 4)) than that in *A*. *thaliana*, the changes in DBI resulted from changes in MGDG content. These data suggest that MGDG was the essential lipid for survival of *C*. *reinhardtii* on cryopreservation.

## Discussion

The cryopreservation of *C*. *reinhardtii* has attracted much attention because of the general importance of resource preservation and this organism being a model alga. The effects of the choice of CPA, rate of lowering temperature, preconditioning, and culture conditions have been investigated intensively [30]. Yet the biochemical basis of these effects remained unclear. In light of the widely accepted idea that the cell membrane is the major injury site at low temperature, insights into changes in membrane lipids during the cryogenic process are of particular significance. We found that cryogenic processes, except for cooling (25°C to −55°C at a rate of 1°C/min) and thawing at 35°C, cause membranes to deteriorate. Cooling leads to a remodelling of membrane lipids, which promotes membrane integrity. Quick thawing at 35°C promotes lipid synthesis and that might accelerate the recovery of *C*. *reinhardtii*. Culture medium plus 5% DMSO played no role in the protection of membranes. In contrast, culture medium plus 5% MeOH attenuated the degradation of membrane lipids and this attenuation increased during cooling. Culture medium plus 5% MeOH also caused increases in total lipid content. The combined effects of culture medium plus 5% MeOH and cooling on lipid remodelling maintain membrane integrity and thus account for the successful cryopreservation of *C*. *reinhardtii*. An increase in MGDG and decrease in DAG is related closely to the survival rate. An interesting finding was that DAG increased dramatically as other lipids degraded, but the increase in DAG did not result from the decrease in the other lipids. PLD and its product PA played no role in lipid metabolism during cryogenic processes. Our findings provide new insights into the cryopreservation of, and lipid metabolism in, algae. Such insights will be helpful for understanding the biochemical processes that occur during deep freezing and for improving cryogenic treatments.

The protective effects of MeOH during cryopreservation are widely accepted. MeOH permeates cells faster than water [9, 31] and is proposed to play the role of osmolyte [32] and to increase membrane permeability [33]. However, these proposals have not received substantive experimental confirmation. Our study shows that MeOH protects membranes by blocking the lipid degradation that is induced by cryogenic stress. It has been observed in leaf senescence that blocking lipid degradation can sustain membrane integrity and thus attenuate physiological symptoms [22]. In light of the possibility that alcohol interferes with lipid metabolism [34], we suggest that MeOH's cryoprotective effects are due mainly to its remodelling of membrane lipids.

Cooling is the key step when cryopreserving algae [4, 35]. It is thought that lowering the temperature gradually during the cooling process dehydrates cells and prevents the formation of intercellular ice crystals [6]. However, this explanation seems to conflict with the general view that during cryopreservation, immediately immersing tissues into liquid nitrogen is necessary for their cellular vitrification [36, 37]. We found that slow cooling attenuates lipid degradation and seems to play a role in the protection of membranes. Given that lipid changes often occur during chilling (from 10°C to 0°C) and near-zero freezing (from 0°C to −8°C) [16], lowering the temperature gradually during the cooling process might provide cells with an opportunity to remodel their membrane lipids to adapt to deep freezing. In particular, MeOH may use this slow process to play its role in blocking the lipid degradation that is induced by deep freezing. Consequently, combining the effects of MeOH and cooling on lipid remodelling sustains membrane integrity and enhances the cryopreservation survival rate of *C*. *reinhardtii* during cryopreservation.

Our study reveals several distinctive characteristics of lipid metabolism in *C*. *reinhardtii*. Firstly, increases in DAG are associated closely with the degradation of other lipids, such as MGDG, but the molecular structure of MGDG does not turnover completely into that of DAG. PLD-mediated PA does not change on freezing. This is different from what occurs in higher plants, where DAG and PA are intermediates of lipid metabolism and lipids are assembled on the glycerol backbone with the same fatty acid chains at *sn*-1 and *sn*-2 positions. Secondly, in culture medium plus 5% MeOH, the total detected lipids increased during the cryogenic process as a whole. Therefore, there must be another class of lipids that serves as an intermediate in lipid metabolism in *C*. *reinhardtii*. Given that the TAG pool can fluctuate in *C*. *reinhardtii* as the environment changes [27, 38], the intermediate could be TAG. We also found previously unreported responses of membrane lipids to deep freezing. For example, freezing in liquid nitrogen seems to cause lipid degradation. However, we think that the degradation does not really occur in the liquid nitrogen itself, but during the subsequent thawing, even though the thawing process only takes minutes.

## Experimental

### Cell Culture Conditions and Measurement of Cell Density

*Chlamydomonas reinhardtii* CC125, which was obtained from the Chlamydomonas Resource Center at the University of Minnesota, was cultured in Tris-acetate-phosphate (TAP) medium [39]. Cells were cultured in a 50-mL flask containing 20 mL of TAP medium and 1 mL was transferred to fresh medium every week. The flasks were shaken on a thermostatic rocking incubator (SPH-211B, Shanghai Shipping, China) at a speed of 120 rpm at 25°C and illuminated continuously with white fluorescent light (PAR = 30 μmol photons m^−2^ s^−1^). Cultures were used for experiment 3–5 days after transfer at a cell density of approximately 0.9–2.0×10^7^ mL^-1^. Cell density was measured by counting with a haemocytometer. Ten percent volume of Lugol's iodine solution was mixed with cell culture (diluted if necessary) and 8 μL of the mixture was used for each chamber. Five small squares in each chamber were counted as a means of calculating the mean number of cells per chamber. The cell number per microliter = mean numbers of cells in five small squares × 5 × 10^4^ × dilution factor.

### CPA Treatment and Cryopreservation Protocols

Stock solutions (10%, v/v) of methanol and DMSO were prepared, using TAP medium as the diluting solution, then packed in 5 mL sterilised Eppendorf tubes and stored at 4°C. The solutions were sterilised using 0.22-μm membrane filters (SLGV033RB, Millex-GV, Ireland). The cryopreservation protocol was based on Crutchfield’s work [4] with minor changes. The cell culture was counted and diluted to a final density of 3.3×10^6^ cells per mL with the CPA (TAP was added when the solution needed to be diluted further) to a total volume of 1.8 mL in 2 mL cryovials (Cat. 5000, Nalgene, Milwaukee, WI). Treatments differed by the type of CPA: culture medium treatment using TAP medium only was the control; in DMSO treatment, DMSO added to a final concentration of 5% (v/v) was the CPA; in MeOH treatment, methanol added to a final concentration of 5% (v/v) was the CPA. The cryovials were placed in a two-compartment freezing container (Cat. 5100 Cryo 1°C Freezing Container, Nalgene, Milwaukee, WI), which contained 250 mL of precooled 4°C isopropanol in the lower compartment to modulate the cooling rate. The 2 mL cryovials containing 1.8 mL cell culture plus CPA were placed in the upper compartment of the freezing container. These steps were carried out at room temperature in the dark. The freezing container was placed in a freezer at −80°C and left undisturbed for 1.5 h (the "Cooling" phase in Fig 1). The cryovials were then removed from the freezing container and put into liquid nitrogen immediately, where they were stored for 1 day ("Freezing in LN" in Fig 1). The cryovials were removed from the liquid nitrogen then quickly put into a water bath at 35°C for 5 min ("Thawing" in Fig 1). The cryovials were centrifuged at 1000 × *g* for 2 min. Then the liquid was discarded and 1 mL of fresh TAP was added to the cryovials for culturing. The cap of the cryovials was loosened and their contents mixed twice a day. The culture conditions were the same as in the Control phase without shaking ("Recovery" in Fig 1). Each treatment was repeated five times for lipid extraction.

The Evans Blue dye test is used to detect the integrity of membranes. The percentage of cells excluded by the test reflects the viability of cultures after cryopreservation [4, 40, 41]. The method that was followed in applying the Evans Blue dye test was as specified by Crutchfield [4]. Equal volumes of Evans Blue dye (0.1% w/v in water) and cell culture (diluted if needed) were mixed and left undisturbed in an Eppendorf tube for 5 min. About 8 μL of the mixture was added to the haemocytometer to enable counting under a microscope (Olympus CX31). To determine whether the cells had taken up the Evans Blue dye, at least 200 were examined at 400× magnification, twice for each sample. The survival rate was calculated as the ratio of the number of cells that retained their original green colour to the total number of cells. Each treatment was performed five times.

### Lipid Extraction and Profiling

Lipids were extracted, samples analysed, and data processed as described previously, with some modifications [16, 42]. In brief, the algal cultures were centrifuged at 1000 × *g* for 2 min. Following this, the liquid was discarded and 1 mL of preheated (75°C) isopropanol containing 0.01% butylated hydroxytoluene was added. The mixture was incubated at 75°C for 15 min. Then 0.8 mL water and 2 mL chloroform:methanol (1:1) were added and the mixture shaken overnight. Then, 1 mL chloroform and 1 mL water were added and the mixture shaken for 2 h, then centrifuged at 1000 × *g* for 5 min. Then the upper layer was extracted with 1 mL chloroform twice more, with 2 h of shaking each time. The lipid samples were examined using electrospray ionization tandem mass spectrometry (ESI-MS/MS). Two internal standards were used to quantify the lipids in each class. The data were profiled as described previously [16].

Five replicates from each treatment were analysed. The total amount of lipid in each class of head group was examined by Q-test and the data from discordant samples were removed [16]. The data were analysed by one-way analysis of variance with Statistical Product and Service Solutions (SPSS) 13.0 software. Statistical significance was determined using the Waller-Duncan method. The DBI was calculated using the formula DBI = (Σ [N × mol% lipid])/100, where N is the number of double bonds in each lipid molecule [43]. The acyl chain length (ACL) was calculated using a formula that was derived from the DBI calculation: ACL = (Σ [NC × mol% lipid])/100, where NC is the number of carbon atoms in each lipid molecule [24].

## Supporting Information

S1 Fig *In vitro* assay of PLD hydrolysis activity in *C*. *reinhardtii*. Thin-layer chromatography analysis of PA from PLD-mediated hydrolysis of PC. CK: negative control, reaction solution without protein addition. AT1-2: protein extracted from *Arabidopsis thaliana* as a positive control. CR1-4: protein extracted from routinely cultured *C*. *reinhardtii* strain CC125. Protein extraction and assays of PLD activity followed the previous procedure [24], with minor changes. *C*. *reinhardtii* (3 mL) in culture medium was centrifuged at 1000 × *g* for 2 min, then ground into fine powder in liquid nitrogen. The *A*. *thaliana* leaves were ground into a fine powder in liquid nitrogen. The powder was placed in a solution that contained 0.5 mL of homogenization buffer (50 mM Tris-HCl/pH 7.5, 10 mM KCl, 1 mM EDTA, 2 mM dithiothreitol, and 0.5 mM phenylmethylsulfonyl fluoride), then mixed and centrifuged at 4800 × *g* for 10 min at 4°C. The supernatant contained the total soluble proteins, the amount of which was determined by following the manufacturer's instructions (Bio-Rad). Thirty micrograms of total protein were added to the transphosphatidylation reaction mixture present at final concentrations of 100 mM MES (pH 6.5), 25 mM CaCl_2_, 0.5 mM SDS, 1% (v/v) ethanol, and 2 mM phosphatidylcholine (PC; from egg yolk), in a total volume of 150 μL. The mixture was placed in a water bath at 30°C and shaken at 100 rpm for 30 min. The reaction was stopped by adding 1 mL chloroform:methanol (2:1) with 0.01% butylated hydroxytoluene. The lipids were extracted and examined by thin-layer chromatography [44].
(TIF)

S1 File Percentage composition (mol%) and amount (nmol per 10 million cells) of all lipid species detected. Five replicates of each treatment for each CPA were carried out and analysed by mass spectrometry. Highlighted values were the discordant data (Q-test). “ave” = average, “stdev” = standard deviation.
(XLSX)

## Abbreviations

ACL: acyl chain length
C: Control (normal growth)
CC’LT: Control-Cooling-freezing in Liquid nitrogen and Thawing
CC’LTR: Control-Cooling-freezing in Liquid nitrogen-Thawing and Recovery growth
CC’T: Control-Cooling and Thawing
CLT: Control-freezing in Liquid nitrogen and Thawing
CC’LR: Control-Cooling-freezing in Liquid nitrogen-maintenance at room temperature for 30 minutes and Recovery growth
CPA: cryoprotective agent
CM: culture medium
DAG: diacylglycerol
DBI: double bond index
DGDG: digalactosyldiacylglycerol
DGTS: diacylglyceryl-N,N,N-trimethylhomoserine
DMSO: dimethyl sulfoxide
ESI-MS/MS: electrospray ionization tandem mass spectrometry
LN: liquid nitrogen
MeOH: methanol
MGDG: monogalactosyldiacylglycerol
PA: phosphatidic acid
PC: phosphatidylcholine
PE: phosphatidylethanolamine
PG: phosphatidylglycerol
PI: phosphatidylinositol
PLD: phospholipase D
PS: phosphatidylserine
SPSS: Statistical Product and Service Solutions
TAG: triacylglycerol
TAP: Tris-acetate-phosphate
