# Supplementary figures and images for: Methanol-Promoted Lipid Remodelling during Cooling Sustains Cryopreservation Survival of Chlamydomonas reinhardtii
Source: PLoS One. 2016 Jan 5;11(1):e0146255. doi: 10.1371/journal.pone.0146255 (PMC4711583; doi:10.1371/journal.pone.0146255)

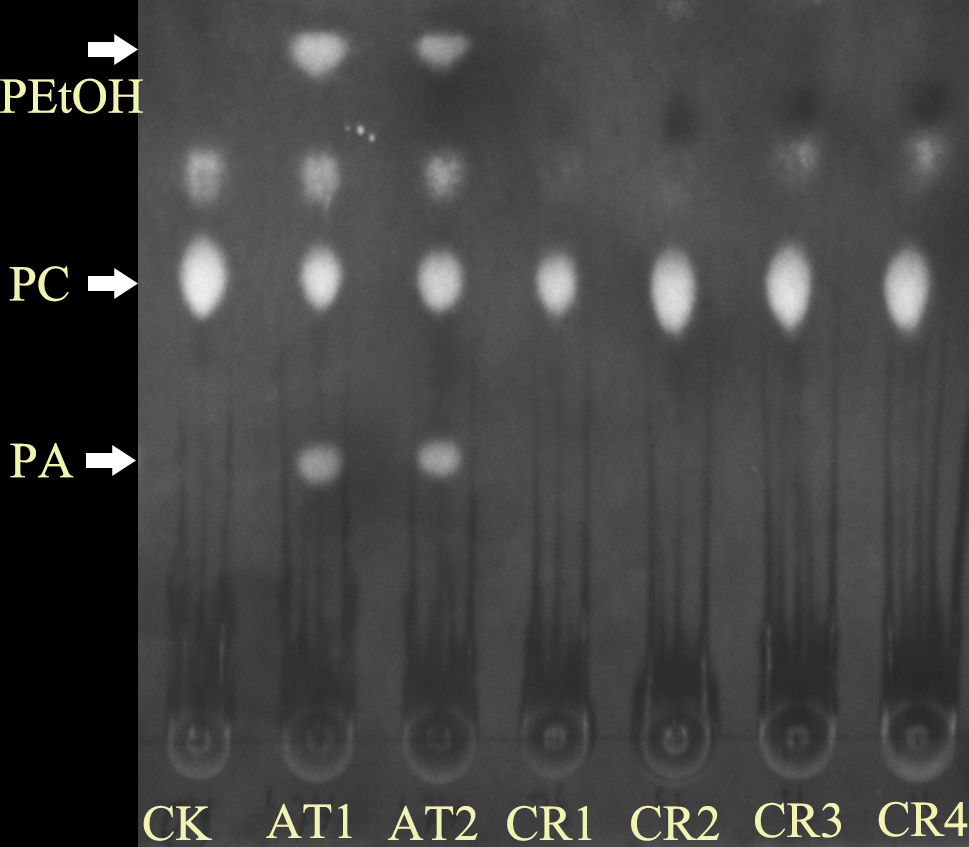

Supplement: S1 Fig — Thin-layer chromatography analysis of PA from PLD-mediated hydrolysis of PC. CK: negative control, reaction solution without protein addition. AT1-2: protein extracted from Arabidopsis thaliana as a positive control. CR1-4: protein extracted from routinely cultured C. reinhardtii strain CC125. Protein extraction and assays of PLD activity followed the previous procedure [24], with minor changes. C. reinhardtii (3 mL) in culture medium was centrifuged at 1000 × g for 2 min, then ground into fine powder in liquid nitrogen. The A. thaliana leaves were ground into a fine powder in liquid nitrogen. The powder was placed in a solution that contained 0.5 mL of homogenization buffer (50 mM Tris-HCl/pH 7.5, 10 mM KCl, 1 mM EDTA, 2 mM dithiothreitol, and 0.5 mM phenylmethylsulfonyl fluoride), then mixed and centrifuged at 4800 × g for 10 min at 4°C. The supernatant contained the total soluble proteins, the amount of which was determined by following the manufacturer's instructions (Bio-Rad). Thirty micrograms of total protein were added to the transphosphatidylation reaction mixture present at final concentrations of 100 mM MES (pH 6.5), 25 mM CaCl2, 0.5 mM SDS, 1% (v/v) ethanol, and 2 mM phosphatidylcholine (PC; from egg yolk), in a total volume of 150 μL. The mixture was placed in a water bath at 30°C and shaken at 100 rpm for 30 min. The reaction was stopped by adding 1 mL chloroform:methanol (2:1) with 0.01% butylated hydroxytoluene. The lipids were extracted and examined by thin-layer chromatography [44]. (TIF) [file pone.0146255.s001.tif]
